# Absence of Serological Evidence of Exposure to *Treponema pallidum* among Children Suggests Yaws Is No Longer Endemic in Kiribati

**DOI:** 10.4269/ajtmh.18-0799

**Published:** 2019-02-04

**Authors:** Becca L. Handley, Robert Butcher, Raebwebwe Taoaba, Chrissy h Roberts, Anasaini Cama, Andreas Müeller, Anthony W. Solomon, Rabebe Tekeraoi, Michael Marks

**Affiliations:** 1 Clinical Research Department, London School of Hygiene & Tropical Medicine, London, United Kingdom;; 2 Eye Department, Ministry of Health and Medical Services, South Tarawa, Kiribati;; 3 The Fred Hollows Foundation, Sydney, Australia;; 4 The International Agency for the Prevention of Blindness, Melbourne, Australia;; 5 Department of Control of Neglected Tropical Diseases, World Health Organization, Geneva, Switzerland;; 6 Centre for Eye Research Australia, University of Melbourne, Melbourne, Australia

## Abstract

Yaws is a neglected tropical disease targeted for eradication by 2020. Kiribati, a Pacific Island nation, was previously endemic for yaws but lacks recent data from which its current endemicity status could be determined. This study tested antibody responses to *Treponema pallidum* to determine if transmission of yaws is taking place among children in Kiribati. Using a commercially available *T. pallidum* particle agglutination kit (Serodia^®^, Fujirebio Inc., Tokyo, Japan), we tested dried blood spots, collected during population-based trachoma prevalence surveys on Tarawa Atoll and Kiritimati Island, for long-lived treponemal antibodies. Dried blood spots from 1,420 children aged 1–9 years were tested. Only two were positive, suggesting *T. pallidum* is not being widely transmitted among children in the settings sampled. These data require support from additional surveys to demonstrate the absence of clinical signs of disease and molecular evidence of infection, to confirm that yaws is no longer endemic in Kiribati.

Yaws is a chronic disease that affects the skin, bones, and cartilage. It is caused by infection with *Treponema pallidum* subsp. *pertenue*. Yaws used to be endemic across the tropics, before a 1950s WHO/United Nations International Children’s Emergency Fund (UNICEF) campaign used parenteral penicillin to produce a ∼95% reduction in cases globally.^1^ However, because of reduced resources and political commitment, premature discontinuation of the campaign, and a lack of follow-on surveillance, yaws cases subsequently resurged in many countries.^2^ Oral azithromycin has recently been shown to be highly effective in treating yaws.^3^ Although potentially compromised by the emergence of azithromycin resistance in *T. pallidum* subsp. *pertenue*,^4^ mass drug administration (MDA) with azithromycin is now the main tool for a renewed WHO-led yaws eradication drive.^5^

The Pacific is one of the most heavily yaws-endemic regions, with roughly 80% of the known global disease burden found in Papua New Guinea, Solomon Islands, and Vanuatu alone. Kiribati is a Pacific nation with a total population of approximately 110,000 people. Like many countries, it was previously endemic for yaws, but lacks recent data.^6^ Other neglected tropical diseases remain endemic in Kiribati and its yaws endemicity status requires clarification. Before this study, azithromycin MDA had not been previously conducted in Kiribati; it was not known whether the prevalence of active trachoma^7,8^ should be the only metric informing planning on its deployment.

Serological testing is key to the diagnosis of treponemal infections and WHO recommends serological surveys to provide evidence that transmission of yaws has been interrupted.^5^ *Treponema*-specific antibodies, such as those detected by the *T. pallidum* particle agglutination (TPPA) test, remain positive for life following infection, and an absence or very low level of serological reactivity in children is therefore consistent with interruption of yaws transmission. (Congenital syphilis could also cause TPPA positivity in children.) Dried blood spot (DBS) testing has been validated for these assays,^9^ facilitating sero-surveys without venepuncture or transport and storage of serum.

As part of public health–level investigations into trachoma in the Pacific Islands, we collected DBS samples during two population-based prevalence surveys conducted in Kiribati in November 2015 and August 2016. Here, we use those samples to provide evidence as to whether or not yaws transmission is ongoing.

Each survey was designed to estimate the prevalence of the active trachoma sign trachomatous inflammation—follicular (TF) in children aged 1–9 years. The Kiritimati survey was powered to have 95% confidence to detect a TF prevalence of 20% in 1–9-year-olds with an absolute precision of 3%. A total of 221 households were visited; these were selected using compact segment sampling. The number of households selected from each village was proportional to that village’s population size.^8^ The Tarawa survey was powered to estimate an expected TF prevalence in 1–9-year-olds of 10% with absolute precision of 3% at the 95% confidence level. Thirty-six villages were selected from the complete list of villages in the evaluation unit, using a probability-proportional-to-size methodology. Within each village, 30 households were drawn at random from a hat.

In both sites, the clinical examination and sample collection and handling followed the same methodology, described elsewhere.^8^ In Kiritimati, fingerprick blood samples were only collected from those aged 1–9 years, whereas in Tarawa, all consenting participants aged ≥ 1 year had fingerprick blood collected.

Dried blood spot samples were tested as previously described.^9^ Briefly, one spot from each selected subject’s card was eluted to a 1:25 dilution in phosphate buffered saline with 0.05% tween80 and tested without technical replicates, using the DBS-modified TPPA assay (Serodia^®^, Fujirebio Inc., Tokyo, Japan). Samples were reported as positive, negative, or indeterminate. All results were reviewed and agreed by two independent researchers (B. H. and M. M.). Indeterminate results were not retested because of the limited number of DBSs available for this study. Positive controls from previous surveys^10^ and manufacturer’s positive and negative controls were included in each run and gave expected results. To determine seroprevalence estimates, 35 indeterminate results were removed from both the numerator and denominator.

Traditionally, active yaws studies are undertaken in 5 to 14-year-olds because this group harbors the peak prevalence of primary yaws. However WHO recommends testing children aged 1–5 years when assessing for interruption of transmission. We therefore tested all samples from children aged 1–9 years, covering both the recommended age range for evaluating transmission interruption and a slightly older age group, to look for evidence of both current and recent transmission between children. To harness the full epidemiological power of the samples, we also tested an arbitrary selection of at least 60 samples, collected during the Tarawa survey, from each of the following age groups: 10–20 years, 21–30 years, 31–40 years, 41–50 years, and ≥ 51+ years. As secondary analyses, we calculated the seroprevalence for children aged 1–5 and 6–9 years (Kiritimati and Tarawa) and 10–14 and ≥ 15 years (Tarawa only). Statistical analysis was conducted in R 3.4.2 (R Foundation for Statistical Computing, Vienna, Austria). Ethical approval was granted by the London School of Hygiene & Tropical Medicine (6319, 8355, 10136) and the Kiribati Ministry of Health and Medical Services (Kiritimati: November 8, 2015; Tarawa: May 25, 2016). Leaders of each village provided verbal consent for community entry. Adults gave written informed consent and parents/guardians provided written informed consent for children aged < 18 years.

In total, 1,420 children aged 1–9 years (49.8% male) provided DBS samples. Only two individuals in this group had a reactive TPPA test (0.14%; 95% CI: 0.02–0.57%). Both of these individuals were resident on Kiritimati. The seroprevalence in children was 0% (95% CI: 0.00–0.47%) on Tarawa and 0.50% (95% CI: 0.09–2.01%) on Kiritimati. Seroprevalences by age group are shown in Table 1. Overall, these data suggest it is unlikely that yaws transmission is ongoing in Kiribati. This conclusion is supported by routine data: no suspected yaws cases have been reported to the Ministry of Health over the last 5 years (A. Tonganibeia, Personal Communication), although yaws is no longer a notifiable disease. It is unlikely that any study would demonstrate a complete absence of apparent seroreactivity to *T. pallidum*, even in populations in which transmission of yaws had truly been interrupted, because the reported specificity of TPPA performed on DBSs is 99.0% (95% CI: 98.1–99.5%).^9^ The prevalence of seropositivity in children is therefore consistent with a complete absence of true positives and the anticipated number of false positives. Equally, the TPPA assay does not distinguish between immunological responses to the subspecies of *T. pallidum*, so the few reactive results in children could represent congenital syphilis.

**Table 1:** Results of the TPPA assay by age group

Age range	*n*	% Male	TPPA seropositivity, *n* (%, 95% CI)	Survey	Rationale
Primary analysis					
1–9	1,420	49.8	2 (0.1, 0.02–0.57)	Kiritimati and Tarawa	Surveillance for interruption of transmission and age range of peak transmission
Secondary analysis					
1–5	837	52.0	1 (0.1, 0.01–0.77)	Kiritimati and Tarawa	Surveillance for interruption of transmission
6–9	583	46.7	1 (0.2, 0.01–1.11)	Kiritimati and Tarawa	Surveillance for interruption of transmission and age range of peak transmission
10–14	151	55.0	0 (0.0, 0.00–3.09)	Tarawa	Age range of peak transmission
≥ 15+	289	33.6	14 (4.8, 2.78–8.18)	Tarawa	–

TPPA = *Treponema pallidum* particle agglutination.

Similarly, TPPA positivity in 4.8% of adults (≥ 15 years) might represent childhood infection with *T. pallidum* subsp. *pertenue* or be a result of exposure to syphilis (for which no national prevalence data are available). Given the low prevalence of seropositivity among children, we contend that seroreactivity in the lower age divisions of this population was almost certainly generated by exposure to *T. pallidum* subsp. *pallidum*, not *T. pallidum* subsp. *pertenue*. The TPPA assay has a sensitivity of 95.5% (95% CI: 91.3–98.0%), so it is possible the true seroprevalence was higher than observed. The relatively high prevalence of seropositivity in adults gives us confidence that sample handling and processing were unlikely to be responsible for the very low prevalence of reactivity in younger age groups.

Our study has some limitations. First, DBS samples were collected during trachoma surveys in which a skin examination^11^ was not performed, and we therefore lack clinical data on yaws-like lesions. Second, each survey was designed to estimate a TF prevalence of ∼10% to 20% and would have been underpowered to detect an extremely low prevalence of seropositivity to *T. pallidum*, which might be expected in the peri-elimination context. However, because of Kiribati’s small population size, the sample tested was greater than 1% of all residents of the country. Third, we had samples from only three of the 21 inhabited islands; although together Tarawa and Kiritimati support 60% of the national population,^12^ we cannot rule out the existence of higher seroprevalence on islands not represented here.

We made use of DBSs to test a proportion of Kiribati’s population for treponemal antibodies. Dried blood spots are simple to collect in low-resource settings, easy to store, and can act as a sample set for future serological or molecular evaluations. By using samples collected during trachoma surveys, we performed a cost-effective study without further fieldwork, demonstrating the benefits of integrated neglected tropical disease surveys,^13–18^ and in particular, one of several potential synergies between programmes targeting trachoma and yaws.^19^ Determining the yaws status of 84 formerly endemic countries is a priority of the yaws eradication programme^20^; this study provides one example of how that might be performed.

Our data will form a valuable component of the evidence required by Kiribati to achieve certification of local yaws elimination. WHO presently recommends a period of at least 3 years of surveillance plus sero-surveys to demonstrate yaws elimination in countries where specific interventions have been undertaken to interrupt transmission of yaws. WHO recommendations for surveillance in countries whose current endemicity is unknown are pending, but sero-surveys are likely to play a role. Based on our data, ongoing transmission of yaws in Kiribati is highly unlikely.
